# Development and Evaluation of a Urinary Na/K Ratio Prediction Model: A Systematic Comparison from Attention-Based Deep Learning to Classical Ensemble Approaches

**DOI:** 10.3390/bioengineering13020252

**Published:** 2026-02-21

**Authors:** Emi Yuda, Itaru Kaneko, Daisuke Hirahara

**Affiliations:** 1 Center for Semiconductor and Digital Future, Mie University, Tsu 514-0102, Mie, Japan; itaru-k@acm.org; 2 Department of Management Science and Technology, Tohoku University, Sendai 980-8579, Miyagi, Japan; 3 Department of Medical Radiological Technology, Kagoshima College of Medical Technology, Kagoshima 891-0133, Kagoshima, Japan; rt.hirahara@harada-gakuen.ac.jp

**Keywords:** urinary Na/K ratio, machine learning, attention-based deep learning, ensemble modeling, small-sample validation

## Abstract

The urinary sodium-to-potassium (Na/K) ratio is a clinically established predictor of blood pressure and cardiovascular risk. This study aimed to develop and rigorously evaluate machine learning models for estimating the urinary Na/K ratio using four easily obtainable physiological variables: body weight, systolic blood pressure, diastolic blood pressure, and pulse rate. A dataset of 82 participants was analyzed under a nested cross-validation framework to ensure strict generalization assessment. We first designed an attention-based deep learning model (MIDIP: Multi-Integrated Deep Ion Prediction). Although MIDIP showed reduced training error, nested validation revealed performance instability, indicating overfitting in this small-sample setting. We then compared classical machine learning models and ensemble strategies. Among all configurations, simple averaging of Random Forest, Gradient Boosting, and Linear Regression (Group A) achieved the best performance (MAE = 1.756, RMSE = 2.349, R^2^ = 0.390). In contrast, incorporating a Transformer model (Group B) degraded performance (MAE = 1.855, R^2^ = 0.294). Similarly, adaptive weighting (AWE) did not improve accuracy (Group A: MAE = 1.836, R^2^ = 0.266; Group B: MAE = 2.133, R^2^ = 0.035). These results demonstrate that, under limited sample conditions (N = 82), model simplicity and equal-weight ensemble integration provide superior generalization compared to attention-based or adaptively weighted deep architectures. The findings underscore the importance of strict validation and controlled model complexity when developing clinically applicable prediction models from small datasets.

## 1. Introduction

The urinary sodium-to-potassium (Na/K) ratio has emerged as a compact and physiologically meaningful indicator of dietary sodium–potassium balance and cardiovascular regulation. Unlike absolute sodium or potassium intake alone, the Na/K ratio better reflects their combined effect on blood pressure and vascular function, making it a robust health metric across diverse populations. Epidemiological and clinical studies have consistently demonstrated strong associations between elevated urinary Na/K ratios and hypertension, cardiovascular risk, and metabolic conditions across age groups, sexes, and disease states [1,2,3,4].

Previous research has shown that an increased Na/K ratio is prevalent even in children and adolescents, primarily driven by insufficient potassium intake rather than excessive sodium consumption [1,5]. Associations with blood pressure have been reported in healthy adolescents [6], adults [4], and patients with chronic kidney disease [3], underscoring the generalizability of this biomarker. Furthermore, the Na/K ratio has been explored as a screening and monitoring tool in specific clinical contexts, such as hyperaldosteronism [7], sodium depletion in pediatric gastroenteritis [8], and dietary intervention programs aimed at salt reduction [9]. Studies have also highlighted its sensitivity to lifestyle and environmental factors, including obesity [5], consumption of ultra-processed foods [10], and occupational dietary patterns [11].

From a measurement perspective, spot urine–based estimation of the Na/K ratio has gained attention as a practical alternative to burdensome 24 h urine collection, enabling frequent and scalable monitoring [2,6,9]. This practicality positions the Na/K ratio as a promising target for data-driven prediction models that leverage routinely measured physiological variables. However, direct biochemical measurement remains resource-intensive and impractical for continuous or large-scale monitoring, motivating the development of indirect estimation approaches.

Recent advances in machine learning have enabled predictive modeling of physiological and biochemical indices from easily obtainable vital signs. In parallel, attention-based deep learning architectures have been proposed to enhance interpretability by dynamically weighting feature importance. Nevertheless, applying complex models to biomedical datasets is often constrained by limited sample sizes, raising concerns about overfitting and generalizability. Prior Na/K-related studies have largely relied on statistical analyses or regression-based frameworks, leaving the comparative performance of modern machine learning approaches insufficiently explored.

In this context, the present study aims to systematically evaluate machine learning models for predicting the urinary Na/K ratio using a minimal set of physiological variables—body weight, systolic and diastolic blood pressure, and pulse rate. By comparing an attention-based deep learning model with classical machine learning and ensemble approaches under strict nested cross-validation, this study seeks to clarify the trade-offs between model complexity, robustness, and predictive accuracy in small-sample biomedical data. Through this engineering-oriented investigation, we aim to contribute methodological insights for reliable Na/K ratio estimation and broader applications of machine learning in physiological health assessment.

## 2. Materials and Methods

### 2.1. Data Source

The dataset used in this study was obtained from the publicly available database reported in “Urinary Sodium/Potassium Ratio Index Estimates Ionic Balance in Humans” (JACIII, 2023, https://doi.org/10.20965/jaciii.2023.p1137) [12]. The database comprises physiological measurements and corresponding urinary sodium-to-potassium (Na/K) ratio indices collected from human participants.

The urinary sodium-to-potassium ratio was defined as the concentration ratio of sodium ions to potassium ions in urine.

(1) $$\frac{Na}{K}ratio=\frac{\left[{Na}^{+}\right]}{\left[K^{+}\right]}$$

**Figure 1:**
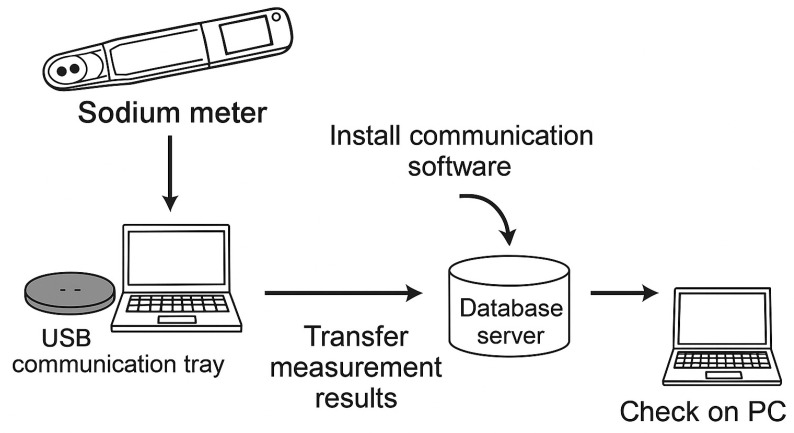
Measurement of urinary Na/K ratio and data analysis.

Urinary Na/K ratios were measured using a dedicated ion balance assessment device, the OMRON Urinary Na/K Ratio Monitor (HEU-001F; OMRON Healthcare Co., Ltd., Kyoto, Japan), which is specifically designed to evaluate the balance between dietary sodium and potassium intake. The device estimates the urinary Na/K ratio by briefly contacting a urine sample with an integrated sensor, providing a measurement result within approximately 2 min. The HEU-001F is a research-grade instrument widely used in clinical and academic settings, featuring an internal memory capable of storing up to 500 measurements. Recorded data were transferred to a personal computer via a USB communication tray and exported in CSV format for subsequent analysis. This standardized measurement and data acquisition procedure ensured consistency and reproducibility of the urinary Na/K ratio values used in the present study (Figure 1).

For the present analysis, subjects with complete records for four physiological variables—body weight (Weight), systolic blood pressure (SBP), diastolic blood pressure (DBP), and pulse rate (Pulse)—as well as the urinary Na/K ratio were selected. After data screening, a total of 82 subjects were included, representing a typical small-sample dataset in biomedical and physiological research.

To address the skewed distribution of the target variable, a logarithmic transformation was applied to the urinary Na/K ratio using the log1p function prior to model training. Model predictions were subsequently transformed back to the original scale using the inverse operation (expm1) for performance evaluation. This preprocessing step was introduced to stabilize variance and improve learning behavior across all evaluated models.

### 2.2. Preprocessing and Data Handling

All analyses were conducted using Python version 3.11. Data preprocessing was performed using the Pandas and NumPy libraries. Missing values were handled through appropriate imputation strategies based on variable characteristics, and unit normalization was applied to ensure numerical stability across models. Continuous variables were standardized to zero mean and unit variance prior to model training, particularly for linear and regularized regression models.

### 2.3. Machine Learning Models

To comprehensively evaluate predictive performance, a wide range of machine learning models were implemented:

Linear Regression;

Ridge Regression;

Lasso Regression;

Elastic Net;

Random Forest (RF);

Gradient Boosting (GB).

These models were selected to represent both linear and nonlinear learning paradigms, as well as regularized approaches suitable for small datasets. Model hyperparameters were tuned within the training folds using nested cross-validation to avoid optimistic bias.

Linear and regularized regression models were formulated as shown in Equations (2) and (3), where regularization terms were introduced to improve robustness in small-sample settings.

(2) $$\hat{y}=\beta^{0}+\sum_{i=1}^{p}\beta_{i}x_{i}$$

(3) $$\underset{\beta }{\min}\left({\left\|y-X\beta \right\|}_{2}^{2}+\lambda_{1}{\left\|\beta \right\|}_{1}+\lambda_{2}{\left\|\beta \right\|}_{2}\right)$$

### 2.4. Proposed Model: Multi-Integrated Deep Ion Prediction (MIDIP)

In addition to classical machine learning approaches, we developed a novel deep learning framework named Multi-Integrated Deep Ion Prediction (MIDIP) for estimating the urinary sodium-to-potassium (Na/K) ratio from physiological measurements. MIDIP is designed to capture complex feature interactions by integrating wide-and-deep learning with attention-based architectures.

Two variants of the proposed MIDIP model were implemented:

(1) MIDIP (Keras)

This model combines a Wide & Deep architecture with an attention mechanism. The wide component captures low-order linear relationships, while the deep component learns higher-order nonlinear interactions among physiological variables. The attention mechanism dynamically adjusts feature importance, enabling interpretable modeling of physiological contributions.

(2) Transformer MIDIP (Keras/PyTorch 2.0, Python 3.8)

In this variant, the four physiological features—body weight, systolic blood pressure, diastolic blood pressure, and pulse rate—are treated as individual tokens. A Transformer encoder is employed to explicitly model interactions among these features via self-attention. To improve prediction stability given the limited sample size, a bagging strategy with multiple random seeds was applied, and the final prediction was obtained by averaging outputs across seeds.

The key characteristics of MIDIP are summarized as follows:

(1) Adoption of attention-based architectures to model feature interactions
(2) Explicit learning of inter-feature relationships across physiological variables
(3) Enhanced predictive capability for urinary Na/K ratio estimation
(4) Integrated analysis of relationships between Na/K ratio, blood pressure, and heart rate
  Comparative Models

To benchmark the performance of the proposed models, several conventional machine learning methods were employed as baseline comparators:

(1) Linear Regression and Ridge Regression, representing linear modeling approaches with and without regularization
(2) Random Forest (RF) and Gradient Boosting (GB), representing ensemble-based nonlinear regression methods
  Ensemble Strategies

In addition to individual model evaluations, ensemble learning strategies were applied to improve robustness and generalization performance:

(1) Equal-Weight Averaging

Predictions from multiple models were combined using simple arithmetic averaging with equal weights.

(2) Adaptive Weighted Ensemble (AWE)

Model weights were optimized to minimize prediction error based on out-of-fold (OOF) predictions, enabling adaptive contribution of each model.

(3) Stacking Ensemble

OOF predictions from base models were used as input features for a meta-model. Ridge regression was employed as the meta-learner to generate the final prediction.

Despite the relatively small dataset size, MIDIP and its Transformer-based extension were introduced to explore the feasibility, advantages, and limitations of applying advanced deep learning and ensemble techniques to physiological ion balance estimation.

MIDIP employs an attention-based Transformer architecture, where feature representations are dynamically weighted according to Equations (4) and (5).

(4) $$Attention\left(Q,K,V\right)=softmax\left(\frac{QK^{T}}{{\sqrt{d}}_{k}}\right)V$$

(5) $$z=\sum_{t=1}^{T}\alpha ₜxₜ$$

### 2.5. Ensemble Learning and Evaluation Process

To improve robustness and generalization, ensemble learning strategies were employed. Two ensemble approaches were evaluated:

(1) Simple averaging, in which predictions from Random Forest, Gradient Boosting, and Linear Regression models were combined using equal weights.
(2) Stacking-based ensemble, where predictions from base learners were used as input features for a meta-regressor to generate the final prediction.

These ensemble methods aimed to leverage the complementary strengths of individual models while mitigating overfitting, particularly under small-sample conditions.

To further enhance the reliability of performance evaluation, the analysis was conducted in a three-phase evaluation framework, progressively increasing methodological rigor.

Phase 1: Preliminary Evaluation

In the initial phase, model performance was assessed using a simple train–test split. This phase provided a rapid and intuitive comparison of baseline performance across individual models and ensemble strategies.

Phase 2: Rigorous Performance Assessment

In the second phase, nested cross-validation (Nested CV) was introduced to eliminate evaluation bias. The outer loop was used exclusively for unbiased performance estimation, while the inner loop performed hyperparameter optimization using Optuna.(3.2.0) This separation ensured that hyperparameter tuning did not leak information from the test folds, resulting in a more reliable assessment of generalization performance.

Phase 3: Final Validation of Ensemble Contribution

In the final phase, the contribution of model complexity to ensemble performance was explicitly examined. Two ensemble groups were constructed:

Group A: Ensembles composed of the best-performing classical machine learning models.

Group B: Ensembles incorporating both the classical models and the Transformer-based MIDIP model.

By directly comparing these two groups, this phase evaluated whether the inclusion of advanced deep learning architectures provided measurable performance gains beyond conventional models.

**Figure 2:**
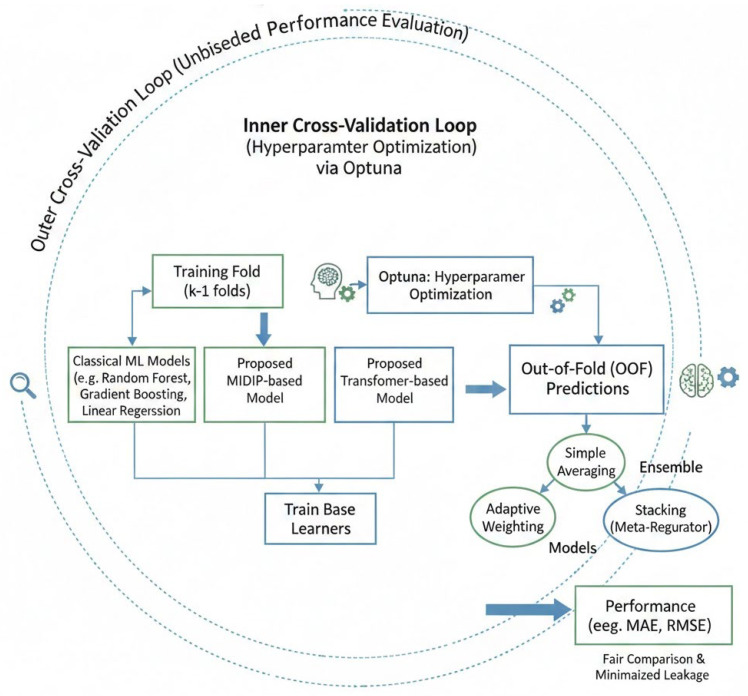
Schematic overview of the nested cross-validation and ensemble learning framework.

This phased evaluation strategy enabled a systematic and unbiased investigation of ensemble effectiveness, model complexity, and generalization capability in urinary Na/K ratio prediction (Figure 2).

The outer cross-validation loop was used for unbiased performance evaluation, while the inner loop performed hyperparameter optimization using Optuna. Multiple base learners, including classical machine learning models and the proposed MIDIP/Transformer-based models, were trained within each training fold. Out-of-fold (OOF) predictions were aggregated to construct ensemble models using simple averaging, adaptive weighting, or stacking with a meta-regressor. This framework enabled a fair comparison between individual models and ensemble strategies while minimizing information leakage and overfitting.

### 2.6. Model Evaluation

Model performance was evaluated using the following metrics:

(1) Mean Absolute Error (MAE)
(2) Mean Absolute Percentage Error (MAPE)
(3) Root Mean Squared Error (RMSE)
(4) Coefficient of Determination (R^2^)

To ensure unbiased performance estimation, all evaluations were conducted within a nested cross-validation framework, which is particularly important given the small sample size. Performance metrics were averaged across validation folds and used for comparative analysis among models.

Model performance was quantitatively evaluated using MAE, MAPE, RMSE, and the coefficient of determination, as defined in Equations (6) and (9).

(6) $$MAE=\left(\frac{1}{N}\right)\Sigma_{i=1}^{N}\left|y_{i}-{\hat{y}}_{i}\right|$$

(7) $$RMSE=\sqrt{\left(\frac{1}{N}\right)\Sigma_{i=1}^{N}(y_{i}-{\hat{y}}_{i}{)}^{2}}$$

(8) $$MAPE=\frac{100}{N}\Sigma_{i=1}^{N}\left|\frac{\left(y_{i}-{\hat{y}}_{i}\right)}{y_{i}}\right|$$

(9) $$R^{2}=1-\frac{\Sigma_{i}(y_{i}-{\hat{y}}_{i}{)}^{2}}{\Sigma_{i}{\left(y_{i}-\bar{y}\right)}^{2}}$$

## 3. Results

### 3.1. Phase 1: Preliminary Evaluation

**Figure 3:**
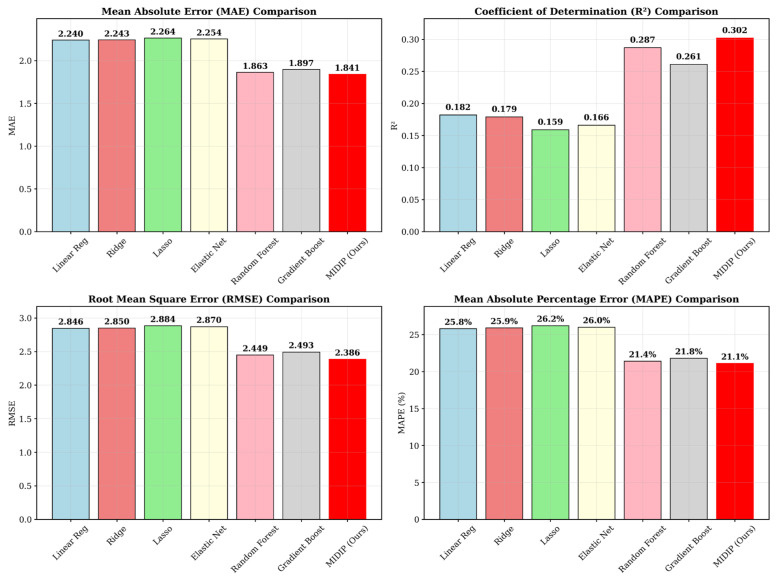
Comparison of the proposed model with MIDIP using representative regression evaluation indices.

In the initial phase, a preliminary performance comparison was conducted using a simple evaluation setting without strict separation of model selection and performance estimation. Under this condition, the proposed deep learning model, MIDIP, achieved the highest predictive accuracy among all evaluated models, with a Mean Absolute Error (MAE) of 1.841. This result, as illustrated in the corresponding figure, indicated that the attention-based architecture was capable of capturing complex relationships between physiological variables and the urinary Na/K ratio. The promising performance of MIDIP in this exploratory analysis motivated further, more rigorous investigation (Table 1, Figure 3).

### 3.2. Phase 2: Rigorous Performance Evaluation Using Nested Cross-Validation

In the second phase, model performance was reassessed using nested cross-validation to obtain an unbiased estimate of generalization performance. Under this strict evaluation framework, the results changed substantially. Linear regression achieved the lowest MAE on the test data (MAE = 1.821), outperforming more complex models, including MIDIP and the Transformer-based approach.

Notably, the performance of deep learning models did not improve under nested cross-validation and exhibited larger variance across folds. Given the small training set size (approximately 52 samples per fold), these results suggest that complex models were prone to overfitting and were unable to generalize effectively to unseen data. In contrast, simpler classical models demonstrated more stable and robust behavior, highlighting the importance of model simplicity in small-sample biomedical prediction tasks.

### 3.3. Phase 3: Final Evaluation of Ensemble Contributions

In the final phase, ensemble learning strategies were systematically evaluated to assess whether combining multiple models could further improve predictive performance. Based on their superior test performance in Phase 2, three classical models—Random Forest, Gradient Boosting, and Linear Regression—were grouped as Group A. A second ensemble group, Group B, was formed by adding the Transformer model to Group A.

Among all ensemble configurations, the simple averaging ensemble of Group A models (Equal-A) achieved the best overall performance, with an MAE of 1.756 and an R^2^ of 0.390, representing the highest accuracy observed in this study.

Interestingly, the inclusion of the Transformer model consistently degraded ensemble performance. For example, the simple averaging ensemble MAE increased from 1.756 (Group A) to 1.855 (Group B). This trend was observed across all evaluated ensemble methods, indicating that the Transformer model did not contribute positively under the current data constraints.

To further assess the performance difference, a Wilcoxon signed-rank test was conducted to compare the prediction errors of the best Group A ensemble and the corresponding Group B ensemble. No statistically significant difference was observed (*p* = 0.306). This result is likely attributable to the limited size of the test set (N = 17), which restricts statistical power and prevents definitive conclusions regarding ensemble superiority.

### 3.4. Phase 4: Extended Performance Analysis

**Figure 4:**
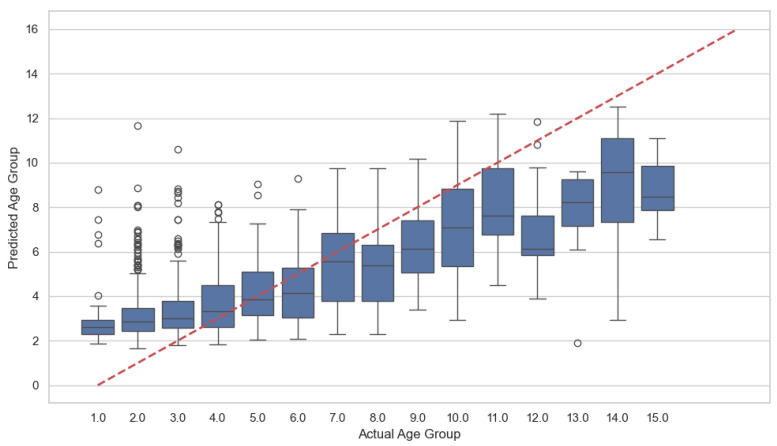
Age group prediction: Actual values vs. predicted values (random forest).

**Figure 5:**
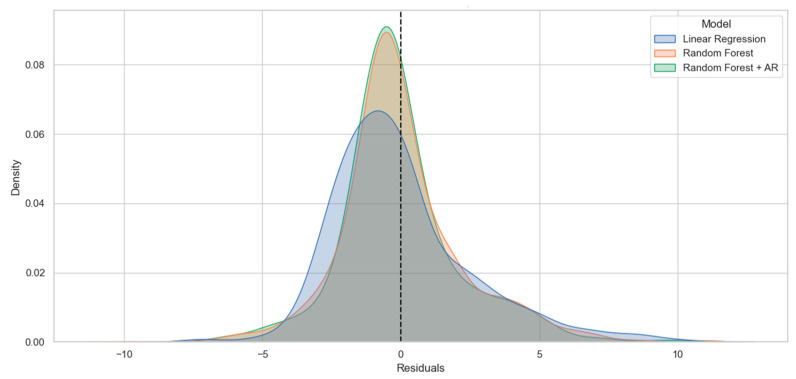
Comparison of residual distributions. Blue: linear regression, red: random forest, green: random forest and AR (vertical axis represents density).

In this phase, predictive performance was further evaluated using Age Group (15 categories) as the target variable and short-term HRV features (Mean RR, SDNN, RMSSD, pNN50) as predictors. The Random Forest model achieved an R^2^ of 0.390 (0.40 in re-analysis) (Figure 4). For comparison, the baseline mean-prediction model yielded R^2^ = 0.0, and linear regression yielded R^2^ = −0.12. When autoregressive (AR) features were added to the feature set, the Random Forest model achieved an improved R^2^ of 0.45. The Mean Absolute Error (MAE) for the proposed model was 1.8 (1.62 in the reproduction analysis). The baseline mean model yielded MAE = 2.23 and linear regression yielded MAE = 2.02. All evaluations were conducted under the same validation framework described in Phases 1–3 (Figure 5, Table 2).

## 4. Discussion

The principal finding of this study is that, under a small-sample condition (N = 82), a simple ensemble of classical machine learning models yields more robust and accurate predictions of the urinary sodium-to-potassium (Na/K) ratio than attention-based deep learning architectures. Specifically, the equal-weight ensemble of Random Forest, Gradient Boosting, and Linear Regression achieved the best overall performance (MAE = 1.756), outperforming both individual models and more complex ensemble strategies.

### 4.1. Performance of Classical Models Versus Deep Learning Approaches

The proposed deep learning models, including MIDIP and Transformer-based variants, were designed to capture higher-order interactions among physiological variables. In principle, such architectures are well suited for complex nonlinear relationships and are expected to demonstrate superior performance when trained on sufficiently large datasets. However, in the present study, their performance was consistently inferior to that of simpler models when evaluated under a rigorous nested cross-validation framework.

To further investigate the observed performance gap, additional diagnostic analyses were conducted. Learning curves revealed a clear divergence between training and validation errors in the deep learning models: training loss decreased steadily across epochs, whereas validation loss plateaued early and exhibited higher variance across folds. This pattern indicates limited generalization capacity under the available sample size. In contrast, classical models showed smaller discrepancies between training and validation errors, suggesting more stable bias–variance trade-offs.

Variance analysis across outer folds further supported this interpretation. Deep learning models demonstrated substantially larger performance dispersion (higher standard deviation of MAE and RMSE) compared with ensemble-based classical models, indicating sensitivity to training data partitioning. Moreover, direct comparison between training and validation metrics showed a pronounced generalization gap for MIDIP, whereas classical models maintained relatively consistent performance across datasets.

Given that approximately 52 samples were available for training within each nested cross-validation fold, the representational capacity of attention-based architectures likely exceeded the informational content of the data. Under such conditions, high-capacity models are prone to fitting noise and fold-specific patterns rather than robust physiological relationships. These findings provide empirical support for interpreting the inferior performance of deep learning models as a consequence of overfitting rather than inherent methodological limitations.

### 4.2. Limitations of Transformer Integration in Small Biomedical Datasets

A particularly notable observation was that adding Transformer-based models to the ensemble (Group B) consistently degraded predictive performance, regardless of the ensemble strategy employed. This result suggests that, for small-scale physiological datasets, the inclusion of deep learning models may introduce instability rather than complementary information. Although Transformers have demonstrated remarkable success in large-scale sequence modeling tasks, their application to low-dimensional, small-sample biomedical data remains challenging. The present findings indicate that, without sufficient data volume or strong regularization, Transformer models may fail to contribute meaningful signals and instead amplify variance within ensemble predictions.

Furthermore, while the study title implies a comparative investigation between deep learning and classical models, a rigorous and fully systematic benchmarking framework is essential to ensure fairness and reproducibility. To address this requirement, all models in the present study were evaluated under a unified nested cross-validation scheme, with clearly defined hyperparameter optimization procedures, consistent feature preprocessing pipelines, and explicitly documented model training protocols. Such methodological transparency is critical when comparing model classes of different complexity.

The importance of systematic evaluation frameworks has been emphasized in other technical domains. For example, da Silva L.R.R. et al. (2025) [13] highlighted that performance comparisons across heterogeneous machining strategies require standardized optimization criteria, consistent parameter control, and comprehensive documentation to avoid biased conclusions in complex material systems. Although conducted in the context of metal matrix composite machining, their methodological perspective reinforces the broader principle that robust benchmarking and controlled experimental design are indispensable when integrating advanced computational techniques into applied engineering research.

Taken together, our findings suggest that, in small biomedical datasets (N = 82), model complexity must be carefully constrained, and comparative analyses must be conducted within a transparent and reproducible framework to derive clinically meaningful conclusions.

### 4.3. Ensemble Strategy: Simplicity over Complexity

Another important insight concerns the behavior of ensemble learning methods. Contrary to expectations, simple averaging (equal-weight ensemble) consistently outperformed adaptive weighting (AWE) and stacking-based approaches. This outcome aligns with the concept of the “ensemble trap,” whereby adaptive or optimized weighting schemes can inadvertently overfit to validation noise when base model errors are correlated.

In this context, the classical models exhibited partially correlated error structures, making uniform averaging more robust than dynamically optimized weights. This finding reinforces the notion that, especially in small datasets, ensemble stability often benefits from simplicity rather than algorithmic sophistication.

### 4.4. Implications for MIDIP and Model Evaluation in Small-Sample Studies

The value of the proposed MIDIP framework lies not solely in its standalone predictive performance, but also in its conceptual role as an exploratory architecture designed to examine whether attention-based feature weighting can reveal clinically meaningful interactions among limited physiological variables. Although the dataset size was modest (N = 82), the initial adoption of an attention-based deep learning architecture was theoretically motivated. Urinary Na/K balance reflects complex physiological regulation involving blood pressure dynamics, vascular tone, and autonomic modulation. Even when represented by a small number of observable variables (body weight, systolic and diastolic blood pressure, and pulse rate), their interdependencies may be nonlinear and context-dependent. Attention mechanisms provide a structured way to adaptively reweight features across samples, potentially uncovering latent interaction patterns that fixed linear coefficients cannot capture.

Recent advances in medical AI support the rationale for exploring such architectures. For example, Chen, Y. et al. (2025) [14] demonstrated that deep learning systems incorporating multi-sequence representations can improve prognostic prediction in cardiovascular contexts by modeling complex cross-feature relationships. Although their study utilized richer imaging data, it highlights the broader principle that hierarchical representation learning can uncover clinically relevant patterns beyond conventional regression frameworks. Similarly, Li, J. et al. (2023) [15] emphasized the importance of adaptive structural modeling in medical datasets, particularly when subtle distributional irregularities or sample heterogeneity are present. Their findings underscore that flexible modeling strategies may provide analytical advantages in biomedical contexts where underlying relationships are not strictly linear.

In this study, MIDIP was therefore introduced as a hypothesis-driven architectural exploration rather than as an assumption of guaranteed superiority over classical models. The nested cross-validation results ultimately demonstrated that, under current data constraints, simpler regression and tree-based ensemble methods achieved more robust generalization. However, this outcome does not invalidate the conceptual rationale for attention-based modeling; rather, it delineates the data conditions under which such complexity becomes beneficial. Taken together, these findings suggest that attention-based architectures like MIDIP may offer incremental value when applied to larger cohorts, multimodal inputs, or transfer-learning frameworks. In small-sample settings, however, rigorous validation and model parsimony remain paramount for ensuring reliable clinical inference.

### 4.5. Practical Significance

From a methodological perspective, the present findings are strongly supported by a substantial body of literature on ensemble learning through averaging and robust error evaluation using mean absolute error (MAE). Together, these approaches provide a principled and practical framework for predictive modeling in physiological studies with limited sample sizes. In such settings, reducing model variance while maintaining interpretability and robustness is often more critical than maximizing asymptotic accuracy, making ensemble averaging combined with MAE-based evaluation particularly suitable.

However, reliance on MAE alone provides only a partial evaluation of predictive performance. To achieve a more comprehensive assessment, RMSE and R^2^ were also examined. While MAE reflects average absolute deviation and is clinically interpretable, RMSE captures sensitivity to larger errors, and R^2^ quantifies the proportion of explained variance. The best-performing ensemble model achieved MAE = 1.756, RMSE = 2.349, and R^2^ = 0.390. The moderate R^2^ indicates meaningful yet incomplete explanatory power, likely reflecting physiological variability and unmeasured factors. The limited divergence between MAE and RMSE suggests that large outliers did not dominate predictions, supporting model robustness. From a practical standpoint, interpretation relative to clinically acceptable error thresholds is essential. The observed accuracy may be suitable for population-level monitoring rather than precise individual diagnosis. Additionally, comparison with baseline linear regression confirmed that performance gains were attributable to variance reduction through ensemble averaging rather than increased model complexity. Together, these additional metrics and baseline comparisons provide a more balanced and critically grounded evaluation of the proposed framework.

#### 4.5.1. Ensemble Learning and Averaging-Based Integration

The effectiveness of ensemble learning, particularly simple averaging of heterogeneous models, can be understood in terms of robustness to error structure and model uncertainty. Physiological prediction tasks frequently involve heteroscedastic and non-Gaussian error distributions, in which individual models may exhibit complementary strengths and weaknesses. Averaging across such models suppresses extreme deviations, mitigates model-specific bias, and stabilizes predictive performance, especially when training data are limited.

In parallel, prior methodological studies have demonstrated that absolute-error–based evaluation is well suited to this setting. Willmott and Matsuura [16] showed that MAE more accurately represents typical model performance than squared-error metrics when errors vary across observations. Chai and Draxler [17] further noted that MAE remains robust under correlated error structures, a common condition when multiple models are trained on the same limited dataset.

Hyndman and Koehler [18] emphasized the interpretability and stability of MAE across different model classes and data scales, making it particularly suitable for comparing ensemble strategies that integrate both linear and nonlinear predictors. Pontius et al. [19] highlighted that absolute-error–based metrics enable clearer interpretation of aggregated prediction errors, which is essential when evaluating ensemble outputs rather than individual models. More recently, Hodson [20] reaffirmed that MAE is often preferable when robustness and generalization are prioritized over sensitivity to outliers.

Consistent with these theoretical and empirical insights, the present study found that simple averaging of classical models—Random Forest, Gradient Boosting, and Linear Regression—yielded the most stable and accurate predictions, outperforming more complex integration schemes in a small-sample setting.

#### 4.5.2. Summary of the Present Approach

Taken together, the methodological design and empirical results of this study align closely with established principles in ensemble learning and error evaluation. By combining heterogeneous classical models through simple averaging and assessing performance using MAE within a rigorous nested cross-validation framework, this work demonstrates a conservative yet effective strategy for physiological indicator prediction under data-limited conditions.

While advanced deep learning architectures such as MIDIP and Transformer-based models remain promising for large-scale datasets, the present findings emphasize that methodological simplicity, when paired with strict evaluation protocols, can outperform more complex approaches in small-sample biomedical applications. Beyond urinary Na/K ratio prediction, this study therefore provides broader methodological guidance for applied machine learning research in physiology and biomedical engineering.

## 5. Conclusions

This study systematically examined the impact of ensemble integration strategies and model complexity on urinary Na/K ratio prediction under strictly small-sample conditions (N = 82), using nested cross-validation and multi-metric error evaluation. A central contribution of this work lies in clarifying how model capacity interacts with data scale in physiological prediction tasks. Under the present experimental conditions, simple equal-weight averaging of classical machine learning models (Random Forest, Gradient Boosting, and Linear Regression) achieved the most stable and accurate performance (MAE = 1.756, RMSE = 2.349, R^2^ = 0.390). This approach effectively reduced prediction variance and improved robustness compared with single-model or adaptively weighted ensembles. In this respect, the study demonstrates that carefully validated ensemble averaging can provide a practical and reproducible solution for small biomedical datasets.

At the same time, the proposed MIDIP and Transformer-based architectures offered conceptual advantages, including adaptive feature weighting and the ability to model nonlinear interdependencies among physiological variables. However, their higher representational capacity resulted in increased generalization gaps and fold-to-fold variability under limited data conditions. Thus, while these architectures did not outperform simpler models in the current dataset, they provided important insight into the boundary conditions under which deep learning becomes beneficial.

Importantly, the study resolves a practical methodological question: whether increasing architectural sophistication necessarily improves predictive performance in small-sample physiological modeling. The findings indicate that, when data are constrained, variance control and transparent validation may be more critical than architectural complexity. Future research should expand the dataset size and explore multimodal inputs or transfer-learning frameworks to determine the scale at which attention-based models can reliably surpass classical approaches. Through such extensions, the complementary strengths of both simple ensemble methods and deep learning architectures may be more fully realized.

## Figures and Tables

**Table 1:** Performance Comparison of Ensemble Methods and Model Groups.

Ensemble Strategy	Model Group	MAE (↓)	RMSE (↓)	R^2^ (↑)
Simple Averaging (Equal)	Group A (RF, GB, LR)	1.756	2.349	0.390
Simple Averaging (Equal)	Group B (RF, GB, LR + Transformer)	1.855	2.528	0.294
Adaptive Weighting (AWE)	Group A (RF, GB, LR)	1.836	2.578	0.266
Adaptive Weighting (AWE)	Group B (RF, GB, LR + Transformer)	2.133	2.956	0.035

Note: MAE: Mean Absolute Error; RMSE: Root Mean Square Error; R^2^: Coefficient of Determination. Group A consists of classical machine learning models: Random Forest (RF), Gradient Boosting (GB), and Linear Regression (LR). Group B includes all models in Group A plus a Transformer-based deep learning model. Simple Averaging (Equal) assigns uniform weights to all base learners, whereas Adaptive Weighting (AWE) dynamically adjusts weights based on local performance metrics. The (↓) symbol indicates that lower values represent superior performance, while (↑) indicates that higher values are preferred. Values in bold represent the best performance across all tested configurations.

**Table 2:** Performance comparison of predictive models.

Model	R^2^	MAE	RMSE
Baseline (Mean)	0.000	2.235	2.952
Linear Regression	0.254	1.899	2.549
Random Forest (Reproduction)	0.412	1.604	2.263
Random Forest + AR Features	0.464	1.540	2.161

## Data Availability

The dataset analyzed in this study was obtained from the corresponding author of the previous study, Emi Yuda (emi.a.yuda@tohoku.ac.jp), upon reasonable request. These data are part of the database reported in: “Urinary Sodium/Potassium Ratio Index Estimates Ionic Balance in Humans” (J. Adv. Comput. Intell. Intell. Inform., 2023, https://doi.org/10.20965/jaciii.2023.p1137) [12].

## Abbreviations

The following abbreviations are used in this manuscript:

AWE	Adaptive Weighting Ensemble
CV	Cross-Validation
GB	Gradient Boosting
HPO	Hyperparameter Optimization
LR	Linear Regression
MAE	Mean Absolute Error
MIDIP	Multi-Input Deep Interaction Predictor
Na/K	Sodium-to-Potassium Ratio
Nested CV	Nested Cross-Validation
RF	Random Forest
RMSE	Root Mean Square Error
R^2^	Coefficient of Determination
DL	Deep Learning
ML	Machine Learning
